# Heparin-Binding Proteins in the Nanoparticle Corona: An Overlooked Player at the Bio–Nano Interface

**DOI:** 10.1021/acsomega.6c09380

**Published:** 2026-10-19

**Authors:** Paulo H. Olivieri, Giselle Z. Justo, Jeffrey D. Esko, Alioscka A. Sousa

**Affiliations:** † Department of Biochemistry, Federal University of São Paulo (UNIFESP), São Paulo, SP 04044-020, Brazil; ‡ Department of Cellular and Molecular Medicine, 8784 University of California, San Diego, California 92093-0687, United States

## Abstract

When nanoparticles (NPs) enter biological environments, they rapidly acquire a layer of adsorbed proteins, known as the protein corona, which plays a central role in their interactions with cells. While extensive work has focused on how corona proteins interact with cell-surface receptors to modulate NP uptake, the role of corona–glycocalyx interactions has received comparatively little attention. In this Minireview, we highlight emerging evidence that heparin-binding proteins (HBPs) within the protein corona can engage glycosaminoglycans, such as heparan sulfate, in the cellular glycocalyx, thereby introducing an additional layer of molecular recognition at the cell surface. Drawing on recently published protein corona data sets, we further show that HBPs are frequently present and often abundant across protein coronas from diverse NP systems. These observations highlight HBPs as important, yet underappreciated, mediators of NP–cell interactions and uptake.

## Graphical abstract

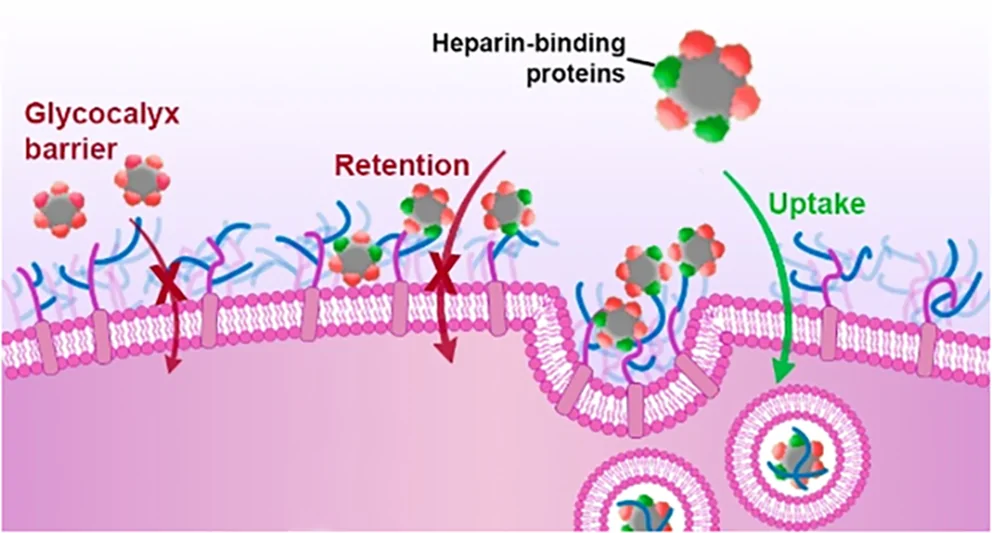

## 1 Introduction

Nanoparticles (NPs) are nanoscale structures widely used as versatile platforms for therapeutic and diagnostic applications. By engineering their physicochemical properties and incorporating bioresponsive features, NPs can enhance the stability, biodistribution, targeting, and delivery of associated cargo molecules to specific cells and tissues.^1,2^ In biological environments such as blood plasma, NPs often acquire a dynamic layer of adsorbed proteins, known as the protein corona, that becomes part of their functional interface.^3,4^ This corona mediates interactions between the NP and the biological environment and, importantly, influences NP–cell interactions, cellular uptake, and ultimately therapeutic delivery.^5–7^

The composition of the protein corona reflects a complex interplay between NP physicochemical properties (e.g., charge, size, shape), the characteristics of the biological fluid (e.g., plasma, serum, healthy vs diseased states), and the exposure conditions (e.g., time, temperature, concentration).^8–10^ In this context, proteomic analyses have been instrumental in identifying and quantifying the hundreds of proteins typically associated with NP protein coronas. These proteins commonly fall into functional classes such as immune recognition, complement, coagulation, acute phase, lipid metabolism, and others.^11^ Among them, heparin-binding proteins (HBPs) form a mechanistically relevant subset, yet they are rarely considered a distinct functional group in studies of the protein corona.

HBPs possess affinity for heparin, a highly sulfated glycosaminoglycan (GAG) with one of the highest negative charge densities among biological molecules. In addition, HBPs can also bind ubiquitous GAGs found at the cell surface, such as heparan sulfate (HS), chondroitin sulfate (CS), and dermatan sulfate (DS).^12–14^ However, despite their prevalence and central roles in cellular processes, HBPs remain underexplored in the context of NP–cell interactions. To address this gap, we begin this Minireview with a brief description of GAGs, the cell glycocalyx, and HBP–GAG interactions. We then discuss potential implications of coronal HBPs for NP uptake and present a quantitative analysis of HBPs within protein coronas based on curated proteomic data sets. We conclude by highlighting research priorities and future directions for understanding the role of coronal HBPs at the bio–nano interface.

## 2 The Cell Glycocalyx and HBP–GAG Interactions

The glycocalyx is a dense layer of carbohydrates that coats the surface of virtually all cells.^15,16^ It forms a highly hydrated and negatively charged meshwork that can extend to hundreds of nanometers from the plasma membrane. HS is a major component of the glycocalyx, occurring as linear, sulfated polysaccharide chains covalently attached to membrane-associated core proteins. These structures form heparan sulfate proteoglycans (HSPGs),^17^ including transmembrane syndecans and GPI-anchored glypicans. Other sulfated GAGs, such as CS and DS, are also present in the glycocalyx attached to proteoglycan cores. In contrast, hyaluronan (HA) is a nonsulfated GAG that is not covalently linked to a protein core. HA can be present as a soluble matrix component or tethered to the membrane via HA-binding receptors such as CD44. Importantly, the composition and architecture of the glycocalyx vary across cell types and tissues and are further modulated by physiological and pathological conditions.^18,19^ This structural and compositional diversity encompasses glycan composition, sulfation patterns, chain length, and overall density and organization. As a result, the glycocalyx can differentially regulate interactions with the surrounding environment.

HSPGs within the glycocalyx provide binding sites for HBPs and serve as key regulators of diverse biological processes,^20–23^ including coagulation, lipid metabolism, growth factor signaling, cell adhesion, and endocytosis. For example, antithrombin (AT) recognizes a highly specific HS pentasaccharide motif, an interaction that activates AT and promotes the inhibition of coagulation proteases;^24^ apolipoprotein E (APOE) binds HS on the hepatocyte surface, a step that facilitates the capture and receptor-mediated clearance of lipoprotein remnants;^25,26^ and fibroblast growth factor 2 (FGF2) binds HS, an interaction that supports FGF2–receptor binding and regulates FGF2 diffusion and spatial distribution within the glycocalyx.^27^ Examples of HBPs binding to GAG classes beyond HS include platelet factor 4 (PF4) binding to CS and DS, as well as APOE binding to CS.^28,29^

At the molecular level, HBP–GAG interactions are initially dominated by electrostatic attraction between the negatively charged sulfate or carboxyl groups of GAGs and clusters of basic lysine or arginine residues on the protein surface. However, binding determinants extend beyond electrostatic attraction. Following initial approach between HBP and GAGs and the ensuing interfacial reorganization, additional forces (such as hydrogen bonding and van der Waals interactions) act across a shape-complementary interface. These contributions, along with entropic effects arising from solvent and ion reorganization, provide the thermodynamic stability and selectivity of the complex.^12,14,30^ Still, aside from the well-defined AT–HS interaction, GAG recognition is generally not dictated by strict sequence specificity but is instead shaped by broader features such as sulfation patterns, three-dimensional charge complementarity and chain flexibility. These characteristics underline the promiscuous binding behavior of many HBPs, which may interact with a broad range of GAG structures across different GAG classes.^21,31^

In the context of the protein corona, HBP–GAG interactions may become substantially more complex. Specifically, the presence and organization of multiple HBP species within the corona may promote multivalent associations with GAG molecules, potentially enhancing GAG-binding affinity and promoting GAG clustering. Conversely, HBP orientation within the corona may conceal GAG-binding sites. Thus, the composition and spatial organization of the corona may influence how HBPs engage with cell-surface GAGs.

## 3 Implications of Coronal HBPs for NP–Cell Interactions

A longstanding paradigm in nanomedicine holds that protein corona composition governs NP–cell interactions and uptake via binding to cell-surface receptors.^7,32–34^ However, the glycocalyx represents the initial cellular interface encountered by incoming NPs, introducing an additional layer of molecular recognition that could modulate NP behavior.^35–39^ For example, the presence of HBPs in the protein corona may enable NPs to interact with cell-surface GAGs, thereby influencing subsequent interactions with membrane receptors or other surface components.

We envision four potential ways in which NP–glycocalyx interactions could influence NP–cell interactions (Figure 1). First, for NPs that lack affinity for GAGs, the glycocalyx may act as a physical barrier, restricting NP penetration and reducing uptake. Second, for NPs capable of binding GAGs, these interactions may serve as anchoring points, promoting NP retention at the cell surface and facilitating subsequent internalization. Third, NP interactions with the glycocalyx could instead retain NPs at the cell surface, thereby delaying or hindering their internalization. Fourth, NPs that bind to cell-surface HSPGs may actively trigger HSPG-dependent entry pathways,^40^ e.g., signaling cascades initiated through HS-modulated receptor tyrosine kinases. Binding to soluble HSPGs could also influence NP uptake. Importantly, these mechanisms are not mutually exclusive and may coexist or occur sequentially during NP–cell interactions.

**Figure 1:**
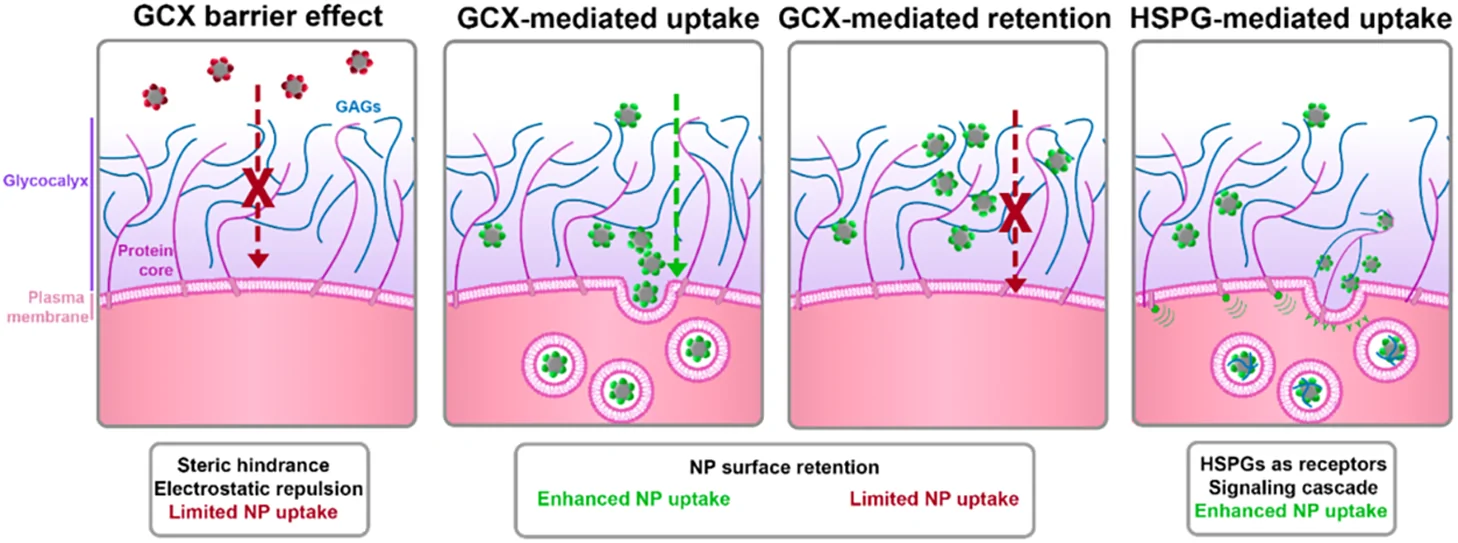
Schematic representation of four potential mechanisms by which the glycocalyx may influence NP uptake. These mechanisms are not mutually exclusive and may coexist or occur sequentially. For simplicity, the glycocalyx (GCX) is depicted as proteoglycans composed of a protein core with covalently attached heparan sulfate chains, while other glycocalyx components are omitted.

Recent experimental findings provide support for some of these ideas. First, we consider studies investigating the influence of the glycocalyx on NP uptake in which coronal HBPs were not specifically addressed. For example, we showed that negatively charged NPs lacking GAG-binding properties, including bare anionic polystyrene NPs and succinylated (anionic) albumin-coated silica NPs, are more efficiently internalized following enzymatic removal of glycocalyx components, consistent with the glycocalyx acting as a barrier to such particles.^35,41^ Several additional reports have similarly shown enhanced NP uptake following glycocalyx degradation or thinning.^42–45^ However, findings from other studies suggest that these effects may be both cell- and NP-type specific.^46^ The glycocalyx can also promote NP uptake, particularly through electrostatic interactions with cationic NPs.^36,47–50^ For example, we showed that NPs coated with aminated (cationic) albumin exhibited significantly higher uptake than NPs coated with unmodified albumin.

Beyond this scenario, the protein corona provides an additional interface for NP interactions with the glycocalyx, particularly through HBPs adsorbed at the NP surface. Notably, such interactions can enhance uptake even for NPs with an overall negative surface charge. For example, we showed that tailored coronas composed of human serum albumin (HSA) supplemented with AT or PF4 led to 5- to 20-fold increases in uptake in CHO cells compared with HSA-coated control particles.^36^ These effects also extended to more complex coronas. Specifically, we demonstrated that a protein corona derived from hyperlipidemic human serum was enriched in HBPs compared to that from normolipidemic serum, leading to an approximately 2-fold increase in NP uptake that appeared to be mediated by corona–glycocalyx interactions. Additionally, we observed similar trends across other cell types, including HeLa cells and human umbilical vein endothelial cells (HUVEC). We also confirmed that these glycocalyx-mediated increases in NP uptake depended not simply on the presence of HBPs in the corona, but on their ability to interact with GAGs, as supported by biophysical measurements. Other groups have reported similar roles for coronal HBPs in NP uptake. For example, Lin et al. showed that PF4 within the protein corona of gold NPs enhanced NP uptake by mouse bEnd.3 endothelial cells via HS-mediated interactions.^49^ The same study further demonstrated that coronal PF4 promoted NP accumulation within the vascular endothelium in vivo. In a separate report, Sato et al. showed that corona–glycocalyx interactions played a key role in the biodistribution and hepatic uptake of lipid NPs (LNPs) in vivo.^51^ Specifically, interactions between APOE-bound LNPs and hepatic HSPGs were found to contribute to rapid blood clearance, accumulation in the space of Disse, and subsequent uptake by hepatocytes. Interestingly, these observations linking corona–GAG interactions to NP uptake were further supported by recent genome-wide screening data reported by Montizaan et al.^52^ In this work, HS was identified as a receptor that promotes uptake of corona-coated silica NPs, alongside classical receptors such as LDLR and SCARB1. Notably, the same work also emphasized that these effects appeared to be NP- and cell-type specific.

Other reports have also associated coronal HBPs with enhanced NP uptake. However, these effects were generally attributed to interactions with membrane receptors, while the potential contribution of the glycocalyx was not specifically addressed. For example, using genome-wide screens, Ngo et al. showed that apolipoprotein B (APOB), together with complement C8, mediated the uptake of serum-coated gold NPs via the LDL receptor in U-87 MG cells.^53^ Similarly, Ge et al. found that APOB-rich coronas were correlated with increased NP uptake in THP-1 macrophages.^54^ Ritz et al. showed that apolipoprotein H (APOH) precoating increased polymeric NP uptake in human mesenchymal stem cells,^55^ while Dilliard et al. showed that it enhanced the uptake of lipid NPs in THP-1 macrophages.^56^ The latter authors also showed that vitronectin (VTN) precoating increased lipid NP uptake in A-498 and U-87 MG cells, presumably through binding to α_v_β_3_ integrin.^56^ In contrast to these findings, other studies have reported that coronal HBPs can decrease NP uptake, although the mechanisms underlying these effects remain unknown. Fedeli et al. found that histidine-rich glycoprotein (HRG) in the corona of silica NPs significantly reduced their uptake by macrophages,^57^ while Aliyandi et al. reported a similar effect of HRG on silica NP uptake by endothelial cells.^5^ Moreover, Schöttler et al. reported that clusterin (CLU) within the protein corona reduced the uptake of PEGylated polystyrene NPs by macrophages,^58^ while Aoyama et al. showed a comparable effect for non-PEGylated silver and silica NPs.^59^ Zhang et al. further showed that CLU precoating reduced the uptake of polystyrene NPs by macrophages, suggesting that this approach could be used to confer a stealth-like behavior to NPs.^60^

Taken together, the current literature indicates that coronal HBPs can influence NP uptake in both positive and negative directions. The mechanisms underlying these effects remain, for the most part, incompletely understood, with the potential involvement of HBP–glycocalyx interactions having received limited attention. Nevertheless, recent studies have begun to investigate these interactions, with evidence suggesting that they can lead to enhanced NP uptake.

## 4 HBPs within NP Protein Coronas

To assess the prevalence and relative abundance of HBPs in protein coronas, we analyzed two recently published protein corona databases: AI2TOX and PROTCROWN (Figure 2A).^61,62^ Together, these resources compile data from 143 independent studies. From these, we selected studies in which coronas were formed using either human serum or human plasma, thus ensuring physiological relevance. Our analysis encompassed 636 individual protein coronas derived from 56 selected studies (Table S1), across which 44 unique proteins were identified as HBPs (Table S2). Relative protein abundance (RPA) values and associated metadata were then extracted from the data sets and used for the descriptive analyses and data visualizations presented below. Further details are provided in the Supporting Information.

**Figure 2:**
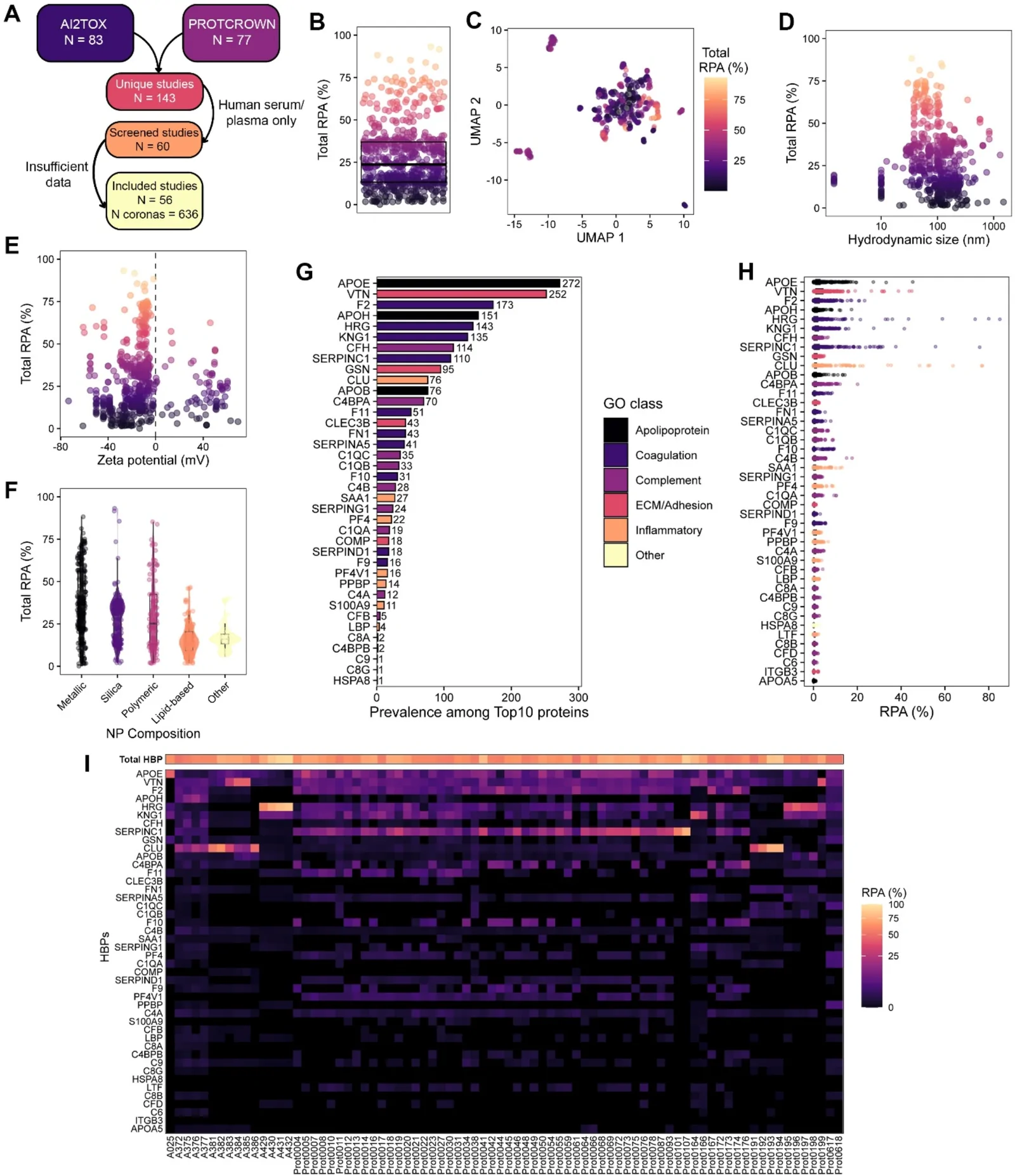
Analysis of HBPs within NP protein coronas. (A) Workflow used to select studies from the AI2TOX and PROTCROWN protein corona databases. (B) Cumulative RPA of HBPs in each corona. (C) UMAP dimensionality reduction of total HBP abundance across protein coronas. (D–F) Cumulative RPA of HBPs as a function of NP properties: (D) size, (E) zeta potential, and (F) core composition. (G) HBPs among the top 10 most abundant corona proteins. Numbers indicate their frequency of appearance. (H) RPA values of individual HBPs across coronas. In both (G) and (H), HBPs are colored according to their GO annotations. (I) Heat map showing the RPA of individual HBPs across coronas (corona IDs shown on the *x* axis). Only coronas in which HBPs account for more than 50% of the total RPA are included. The cumulative RPA of HBPs in each corona is additionally shown as a horizontal color bar. In (G), (H) and (I), proteins are indicated by their gene names.

Across the analyzed coronas, HBPs represented a substantial fraction of the total protein abundance, with a median of 23.6% (IQR, 13.3–37.0%) (Figure 2B). Notably, HBPs accounted for more than 50% of the corona in 13% of the samples, indicating that they were more abundant than non-HBPs in these cases. UMAP dimensionality reduction was used to explore HBP enrichment patterns across coronas, revealing a nonrandom distribution (Figure 2C). HBP abundance was also plotted against NP physicochemical properties, including particle size, zeta potential, and core composition (Figure 2D–F).

The distribution of individual HBPs across the analyzed coronas was further explored in Figure 2G–I. Figure 2G lists the HBPs occurring among the top 10 most abundant proteins, with APOE and VTN appearing in the top 10 in approximately 40% of coronas. The RPA of individual HBPs across coronas is shown in Figure 2H, with several proteins, including APOE, APOB, APOH, VTN, AT (SERPINC1), HRG, KNG1, and CLU, reaching RPA values above 10% in some coronas. The corresponding heat map for selected coronas is presented in Figure 2I, illustrating that high total HBP abundance can result either from a few highly abundant HBPs or from the combined contribution of multiple less abundant HBPs.

Notably, the most prevalent and abundant coronal HBPs identified above have been associated with either increased or decreased NP uptake, as described in the preceding section. At the same time, these HBPs have well-characterized GAG-binding properties and physiological functions that depend, at least in part, on interactions with cell-surface GAGs. Together, these observations point to HBP–GAG interactions as a potentially relevant, yet largely unexplored, aspect of how the protein corona may influence NP biointeractions.

## 5 Conclusions and Outlook

HBPs represent a mechanistically relevant yet largely overlooked component of the protein corona, with the potential to significantly influence NP–cell interactions and NP-based therapeutics more broadly. Here, we highlighted recent experimental evidence indicating that coronal HBPs can enhance NP uptake through interactions with the cellular glycocalyx. Certain HBPs have also been implicated in reducing NP uptake, although the potential contribution of glycocalyx interactions has not been investigated in these cases. We also analyzed recently published protein corona data sets and found that HBPs are frequently present and can represent a substantial fraction of the corona across diverse NP systems.

Moving forward, several key questions remain to be addressed to better understand the role of coronal HBPs in glycocalyx interactions and determine how these interactions may influence NP uptake and in vivo behavior.

- *Corona composition*. Future proteomics studies should incorporate the systematic identification and quantification of HBPs in coronas. HBPs should be considered not only within their conventional functional classifications (e.g., coagulation, complement, apolipoproteins, acute-phase proteins), but also as a distinct functional class based on their capacity to interact with GAGs.
- *Corona–GAG interactions*. Biophysical studies should directly characterize interactions between HBP-containing coronas and GAGs to determine whether adsorbed HBPs retain their GAG-binding capacity and to define relevant binding parameters, including affinity, kinetics (particularly residence time) and avidity. Such measurements should be performed using both soluble and surface-immobilized GAGs, with the latter potentially providing a more physiologically relevant representation of the multivalent glycans encountered at the cell surface.
- *“Mind the GAG” – Cellular uptake*. The influence of coronal HBPs on NP uptake should be investigated not only in the context of their interactions with cellular receptors, but also in the context of their interactions with GAGs within the glycocalyx. Future studies should therefore determine whether HBP–glycocalyx interactions contribute to NP surface retention and influence subsequent internalization.
- *In vivo relevance and disease context*. The influence of corona–glycocalyx interactions on the biological fate and therapeutic performance of NPs in vivo should be investigated. This may be particularly relevant under pathological conditions in which the HBP content of the corona and/or the cell glycocalyx are altered, such as in dyslipidemia, atherosclerosis, and cancer.

Taken together, these considerations underscore the importance of integrating HBPs and GAG biology into current models of NP–cell interactions and into the rational design of nanomedicines. Engineering NP systems that can leverage both corona–glycocalyx and corona–receptor interactions may offer a multidimensional strategy to achieve more selective and efficient NP uptake.

## Supplementary Material



## Notes

Supporting methods, compilation, and analysis of protein corona databases; list of publications included in the analysis (Table S1); and list of curated heparin-binding proteins (Table S2) (PDF)

## References

[1] Germain M., Caputo F., Metcalfe S., Tosi G., Spring K., Åslund A. K., Pottier A., Schiffelers R., Ceccaldi A., Schmid R. (2020). Delivering the power of nanomedicine
to patients today. J. Controlled Release.

[2] Puri S., Mazza M., Roy G., England R. M., Zhou L., Nourian S., Subramony J. A. (2023). Evolution
of nanomedicine formulations
for targeted delivery and controlled release. Adv. Drug Delivery Rev..

[3] Cai R., Chen C. (2019). The crown and the scepter:
roles of the protein corona in nanomedicine. Adv. Mater..

[4] Li H., Wang Y., Tang Q., Yin D., Tang C., He E., Zou L., Peng Q. (2021). The protein
corona and its effects
on nanoparticle-based drug delivery systems. Acta Biomater..

[5] Aliyandi A., Reker-Smit C., Bron R., Zuhorn I. S., Salvati A. (2021). Correlating
corona composition and cell uptake to identify proteins affecting
nanoparticle entry into endothelial cells. ACS
Biomater. Sci. Eng..

[6] Ban Z., Yuan P., Yu F., Peng T., Zhou Q., Hu X. (2020). Machine learning predicts
the functional composition of the protein
corona and the cellular recognition of nanoparticles. Proc. Nat. Acad. Sci. U.S.A..

[7] Walkey C. D., Olsen J. B., Song F., Liu R., Guo H., Olsen D. W. H., Cohen Y., Emili A., Chan W. C. (2014). Protein
corona fingerprinting predicts the cellular interaction of gold and
silver nanoparticles. ACS Nano.

[8] Blume J. E., Manning W. C., Troiano G., Hornburg D., Figa M., Hesterberg L., Platt T. L., Zhao X., Cuaresma R. A., Everley P. A. (2020). Rapid, deep and precise profiling of the plasma
proteome with multi-nanoparticle protein corona. Nat. Commun..

[9] Corbo C., Molinaro R., Tabatabaei M., Farokhzad O. C., Mahmoudi M. (2017). Personalized protein corona on nanoparticles
and its
clinical implications. Biomater. Sci..

[10] Lundqvist M., Augustsson C., Lilja M., Lundkvist K., Dahlbäck B., Linse S., Cedervall T. (2017). The nanoparticle
protein corona formed in human blood or human blood fractions. PloS One.

[11] Cai R., Ren J., Ji Y., Wang Y., Liu Y., Chen Z., Farhadi
Sabet Z., Wu X., Lynch I., Chen C. (2020). Corona of
thorns: the surface chemistry-mediated protein corona perturbs the
recognition and immune response of macrophages. ACS Appl. Mater. Interface.

[12] Meneghetti M. C. Z., Hughes A. J., Rudd T. R., Nader H. B., Powell A. K., Yates E. A., Lima M. A. (2015). Heparan
sulfate and heparin interactions
with proteins. J. R. Soc. Interface.

[13] Esko, J. D. ; Kimata, K. ; Lindahl, U. Proteoglycans and sulfated glycosaminoglycans. In Essentials of Glycobiology; Varki, A. ; Cummings, R. D. ; Esko, J. D. , Eds.; Cold Spring Harbor Laboratory Press: Cold Spring Harbor, 2017.

[14] Alotaibi F.
S., Alsadun M. M., Alsaiari S. A., Ramakrishnan K., Yates E. A., Fernig D. G. (2024). Interactions
of proteins with heparan
sulfate. Essays Biochem..

[15] Möckl L. (2020). The emerging
role of the mammalian glycocalyx in functional membrane organization
and immune system regulation. Front. Cell Dev.
Biol..

[16] Foote C. A., Soares R. N., Ramirez-Perez F. I., Ghiarone T., Aroor A., Manrique-Acevedo C., Padilla J., Martinez-Lemus L. (2022). Endothelial
glycocalyx. Compr. Physiol..

[17] Sarrazin S., Lamanna W. C., Esko J. D. (2011). Heparan
sulfate proteoglycans. Cold Spring Harbor Perspect.
Biol..

[18] Kuo J. C.-H., Gandhi J. G., Zia R. N., Paszek M. J. (2018). Physical
biology
of the cancer cell glycocalyx. Nat. Phys..

[19] Buffone A., Weaver V. M. (2019). Don’t sugarcoat it: How glycocalyx
composition influences cancer progression. J.
Cell Biol..

[20] Tumova S., Woods A., Couchman J. R. (2000). Heparan sulfate proteoglycans on
the cell surface: versatile coordinators of cellular functions. Int. J. Biochem. Cell Biol..

[21] Gómez
Toledo A., Sorrentino J. T., Sandoval D. R., Malmström J., Lewis N. E., Esko J. D. (2021). A Systems View of the Heparan Sulfate
Interactome. J. Histochem. Cytochem..

[22] Perez S., Makshakova O., Angulo J., Bedini E., Bisio A., de Paz J. L., Fadda E., Guerrini M., Hricovini M., Hricovini M. (2023). Glycosaminoglycans: what remains to be deciphered?. JACS Au.

[23] Vallet S. D., Clerc O., Ricard-Blum S. (2021). Glycosaminoglycan–protein
interactions: the first draft of the glycosaminoglycan interactome. J. Cytochem. Histochem..

[24] Rezaie A. R., Giri H. (2020). Anticoagulant and signaling
functions of antithrombin. J. Thromb. Haemostasis.

[25] Libeu C. P., Lund-Katz S., Phillips M. C., Wehrli S., Hernáiz M. J., Capila I., Linhardt R. J., Raffaı R. L., Newhouse Y. M., Zhou F., Weisgraber K. H. (2001). New insights
into the heparan sulfate proteoglycan-binding activity of apolipoprotein
E. J. Biol. Chem..

[26] Mahley R. W., Ji Z.-S. (1999). Remnant lipoprotein
metabolism: key pathways involving cell-surface
heparan sulfate proteoglycans and apolipoprotein E. J. Lipid Res..

[27] Duchesne L., Octeau V., Bearon R. N., Beckett A., Prior I. A., Lounis B., Fernig D. G. (2012). Transport
of fibroblast growth factor
2 in the pericellular matrix is controlled by the spatial distribution
of its binding sites in heparan sulfate. PLoS
Biol..

[28] Lord M. S., Cheng B., Farrugia B. L., McCarthy S., Whitelock J. M. (2017). Platelet
factor 4 binds to vascular proteoglycans and controls both growth
factor activities and platelet activation. J.
Biol. Chem..

[29] Burgess J. W., Liang P., Vaidyanath C., Marcel Y. L. (1999). ApoE of the HepG2
cell surface includes a major pool associated with chondroitin sulfate
proteoglycans. Biochemistry.

[30] Xu D., Esko J. D. (2014). Demystifying heparan
sulfate–protein interactions. Annu. Rev.
Biochem..

[31] Ori A., Wilkinson M. C., Fernig D. G. (2011). A systems biology approach for the
investigation of the heparin/heparan sulfate interactome. J. Biol. Chem..

[32] Dawson K. A., Yan Y. (2021). Current understanding
of biological identity at the nanoscale and
future prospects. Nat. Nanotechnol..

[33] Francia V., Yang K., Deville S., Reker-Smit C., Nelissen I., Salvati A. (2019). Corona composition
can affect the
mechanisms cells use to internalize nanoparticles. ACS Nano.

[34] Lara S., Alnasser F., Polo E., Garry D., Lo Giudice M. C., Hristov D. R., Rocks L., Salvati A., Yan Y., Dawson K. A. (2017). Identification of
receptor binding to the biomolecular
corona of nanoparticles. ACS Nano.

[35] Olivieri
Jr P. H., Assis I. F., Lima A. F., Hassan S. A., Torquato R. J., Hayashi J. Y., Tashima A. K., Nader H. B., Salvati A., Justo G. Z., Sousa A. A. (2024). Glycocalyx Interactions
Modulate the Cellular Uptake of Albumin-Coated Nanoparticles. ACS Appl. Bio Mater..

[36] Olivieri P. H., Hayashi J. Y., Torquato R. J., Lima A. F., Pereira T. P., Lima I. F., Fonseca F. L., Iwai L. K., Nader H. B., Tashima A. K. (2025). Heparin-Binding Proteins
in the Nanoparticle Corona Enhance Cellular Uptake through Glycocalyx
Interactions. ACS Nano.

[37] Fu L., Kim H. N., Sterling J. D., Baker S. M., Lord M. S. (2022). The role
of the cell surface glycocalyx in drug delivery to and through the
endothelium. Adv. Drug Delivery Rev..

[38] Bussin B., MacDuff M. G., Ngo W., Chan W. C. (2025). Cellular Glycocalyx
Affects Nanoparticle Access to Cell Membranes and Uptake. Adv. Mater..

[39] Peter B., Kanyo N., Kovacs K. D., Kovács V., Szekacs I., Pécz B., Molnár K., Nakanishi H., Lagzi I., Horvath R. (2023). Glycocalyx
Components
Detune the Cellular Uptake of Gold Nanoparticles in a Size-and Charge-Dependent
Manner. ACS Appl. Bio Mater..

[40] Christianson H. C., Belting M. (2014). Heparan sulfate proteoglycan
as a cell-surface endocytosis
receptor. Matrix Biol..

[41] Olivieri P. H., Jesus M. B., Nader H. B., Justo G. Z., Sousa A. A. (2022). Cell-surface
glycosaminoglycans regulate the cellular uptake of charged polystyrene
nanoparticles. Nanoscale.

[42] Möckl L., Hirn S., Torrano A. A., Uhl B., Bräuchle C., Krombach F. (2017). The glycocalyx regulates
the uptake of nanoparticles
by human endothelial cells in vitro. Nanomedicine.

[43] Cheng M. J., Kumar R., Sridhar S., Webster T. J., Ebong E. E. (2016). Endothelial
glycocalyx conditions influence nanoparticle uptake for passive targeting. Int. J. Nanomed..

[44] Bridges C. A., Fu L., Yeow J., Huang X., Jackson M., Kuchel R. P., Sterling J. D., Baker S. M., Lord M. S. (2025). The interplay between
endothelial glycocalyx maturity and both the toxicity and intracellular
uptake of charged nanoparticles. Acta Biomater..

[45] Cheng M. J., Bal N. N., Prabakaran P., Kumar R., Webster T. J., Sridhar S., Ebong E. E. (2019). Ultrasmall
gold nanorods: synthesis
and glycocalyx-related permeability in human endothelial cells. Int. J. Nanomed..

[46] Kampen L., Remmo A., Twamley S. G., Weller A., Stach A., Turko P., Löwa N., Wiekhorst F., Ludwig A. (2024). Rapid cellular uptake of citrate-coated iron oxide
nanoparticles unaffected by cell-surface glycosaminoglycans. Nanoscale Adv..

[47] Liu Y., He Y., Xu H., Remmo A., Wiekhorst F., Heymann F., Liu H., Schellenberger E., Häckel A., Hauptmann R. (2024). The Role of Glycocalyx
Diversity and Thickness for Nanoparticle Internalization in M1-/M2-like
Macrophages. Nano Lett..

[48] Zhou R., Zhou H., Xiong B., He Y., Yeung E. S. (2012). Pericellular
matrix enhances retention and cellular uptake of nanoparticles. J. Am. Chem. Soc..

[49] Lin Z. P., Ngo W., Mladjenovic S. M., Wu J. L., Chan W. C. (2023). Nanoparticles
bind to endothelial cells in injured blood vessels via a transient
protein corona. Nano Lett..

[50] Paoloni J., Jackson M., Pandzic E., Alam M. S., Meijering E., Yeow J., Lord M. S. (2026). Decoding
Endothelial Glycocalyx-Nanoparticle
Interactions via a Systematic Polymeric Nanoparticle Library and High-Content
Characterization. Small.

[51] Sato Y., Kinami Y., Hashiba K., Harashima H. (2020). Different
kinetics for the hepatic uptake of lipid nanoparticles between the
apolipoprotein E/low density lipoprotein receptor and the N-acetyl-d-galactosamine/asialoglycoprotein
receptor pathway. J. Controlled Release.

[52] Montizaan D., Bartucci R., Reker-Smit C., de Weerd S., Åberg C., Guryev V., Spierings D. C., Salvati A. (2024). Genome-wide forward
genetic screening to identify receptors and proteins mediating nanoparticle
uptake and intracellular processing. Nat. Nanotechnol..

[53] Ngo W., Wu J. L., Lin Z. P., Zhang Y., Bussin B., Granda Farias A., Syed A. M., Chan K., Habsid A., Moffat J., Chan W. C. W. (2022). Identifying cell receptors for the
nanoparticle protein corona using genome screens. Nat. Chem. Biol..

[54] Ge Y., Fu F., Gao Y., He T., Mailänder V., Crespy D., Landfester K., Jiang S. (2025). Physiological pH Transition-Driven
Protein Corona Dynamics Regulate Cellular Uptake and Inflammatory
Responses of Silica Nanoparticles. Adv. Sci..

[55] Ritz S., Schöttler S., Kotman N., Baier G., Musyanovych A., Kuharev J., Landfester K., Schild H., Jahn O., Tenzer S., Mailänder V. (2015). Protein corona of nanoparticles:
distinct proteins regulate the cellular uptake. Biomacromolecules.

[56] Dilliard S. A., Cheng Q., Siegwart D. J. (2021). On the
mechanism of tissue-specific
mRNA delivery by selective organ targeting nanoparticles. Proc. Nat. Acad. Sci. U.S.A..

[57] Fedeli C., Segat D., Tavano R., Bubacco L., De Franceschi G., de Laureto P. P., Lubian E., Selvestrel F., Mancin F., Papini E. (2015). The functional dissection of the
plasma corona of SiO 2-NPs spots histidine rich glycoprotein as a
major player able to hamper nanoparticle capture by macrophages. Nanoscale.

[58] Schöttler S., Becker G., Winzen S., Steinbach T., Mohr K., Landfester K., Mailänder V., Wurm F. R. (2016). Protein adsorption is required for
stealth effect of
poly (ethylene glycol)-and poly (phosphoester)-coated nanocarriers. Nat. Nanotechnol..

[59] Aoyama M., Hata K., Higashisaka K., Nagano K., Yoshioka Y., Tsutsumi Y. (2016). Clusterin in the protein
corona plays a key role in
the stealth effect of nanoparticles against phagocytes. Biochem. Biophys. Res. Commun..

[60] Zhang X., Si S., Lieberwirth I., Landfester K., Mailänder V. (2025). Engineered
protein corona sustains stealth functionality of nanocarriers in plasma. J. Nanobiotech..

[61] Canchola A., Li K., Chen K., Borboa-Pimentel A., Chou C., Dela Rama R., Chen C.-Y., Chen X., Strobel M., Riviere J. E. (2025). Meta-analysis and machine
learning prediction of protein corona composition
across nanoparticle systems in biological media. ACS Nano.

[62] Su Y., Fu X., Wei L., Zhang Z., Zou Q., Cui F. (2025). PROTCROWN:
A Manually Curated Resource of Protein Corona Data for Unlocking the
Potential of Protein–Nanoparticle Interactions. Nano Lett..

