# Supplementary material for: Heparin-Binding Proteins in the Nanoparticle Corona: An Overlooked Player at the Bio–Nano Interface
Source: ACS Omega. 2026 Oct 19;11(43):66016–22. doi: 10.1021/acsomega.6c09380 (PMC13736882; doi:10.1021/acsomega.6c09380)
Supplement: Supplementary file 1 [file ao6c09380_si_001.pdf]

Supplementary Information

Heparin-Binding Proteins in the Nanoparticle Corona: An Overlooked Player at the Bio–Nano Interface

Paulo H. Olivieri Jr.,[1,2,*] Giselle Z. Justo,[1] Jeffrey D. Esko,[2] Alioscka A. Sousa[1,*]

[1] Department of Biochemistry, Federal University of São Paulo (UNIFESP), São Paulo, SP 04044-020, Brazil

[2] Department of Cellular and Molecular Medicine, University of California, San Diego, CA 92093-0687, United States

* alioscka.sousa@unifesp.br
* p.olivierijr@hotmail.com

Dataset compilation and analysis

Protein corona datasets were retrieved from the AI2TOX [1] and PROTCROWN [2] databases, which collectively comprise 143 published studies. Studies were considered eligible when protein coronas were formed using human plasma or serum and quantitative protein-level data were available. Of the 60 studies initially identified as eligible, four were excluded because the available data were insufficient for quantitative analysis. The final dataset comprised 636 individual protein coronas from 56 studies (**Table S1**).

Protein identifiers were standardized to their corresponding human gene symbols. For each corona, we then screened proteins for known HBPs using Gene Ontology (GO) annotations from AmiGO 2 [3,4] together with experimentally validated protein–heparin interactions curated in IntAct [5]. This was complemented by a literature search to identify additional HBPs with experimental evidence of binding to heparin or HS. In total, 44 unique proteins were identified as HBPs across all coronas (**Table S2**).

Next, we compiled the relative protein abundance (RPA) of each HBP in each corona. When RPA values were not directly reported (19 coronas), they were calculated from the available spectral counts according to:

$$\mathrm{RPA}(\%) = 100 \times \frac{\text{Spectral count (protein)}}{\text{Total spectral counts}}$$

The resulting RPA values, together with associated metadata, were used for all descriptive analyses and data visualizations presented in the main text. The analyses were exploratory and descriptive, and no formal statistical testing was performed.

Table S1. List of the 56 publications included in the final analysis.

#	Title	Database	PMID	DOI
1	Correlating Corona Composition and Cell Uptake to Identify Proteins Affecting Nanoparticle Entry into Endothelial Cells	AI2TOX	34761907	10.1021/acsbiomaterials.1c00804
2	Impact of protein pre-coating on the protein corona composition and nanoparticle cellular uptake	AI2TOX	26513421	10.1016/j.biomaterials.2015.10.019
3	Nano-bio interface between human plasma and niosomes with different formulations indicates protein corona patterns for nanoparticle cell targeting and uptake	AI2TOX	33666624	10.1039/D0NR07229J
4	Corona Composition Can Affect the Mechanisms Cells Use to Internalize Nanoparticles	AI2TOX	31525954	10.1021/acsnano.9b03824
5	Plasma proteins facilitates placental transfer of polystyrene particles	AI2TOX	32907583	10.1186/s12951-020-00676-5
6	Peptide ligands on the PEGylated nanoparticle surface and human serum composition are key factors for the interaction between immune cells and nanoparticles	AI2TOX	36343480	10.1016/j.colsurfb.2022.112981
7	Protein Corona of Nanoparticles: Distinct Proteins Regulate the Cellular Uptake	AI2TOX	25794196	10.1021/acs.biomac.5b00108
8	Denaturation via Surfactants Changes Composition of Protein Corona	AI2TOX	29660271	10.1021/acs.biomac.8b00278
9	Biomolecular Corona Stability in Association with Plasma Cholesterol Level	AI2TOX	35957093	10.3390/nano12152661
10	Brain Targeting by Liposome–Biomolecular Corona Boosts Anticancer Efficacy of Temozolomide in Glioblastoma Cells	AI2TOX	30015470	10.1021/acschemneuro.8b00339
11	Timing of Heparin Addition to the Biomolecular Corona Influences the Cellular Uptake of Nanocarriers	AI2TOX	31449399	10.1021/acs.biomac.9b00777
12	Influence of Poly(ethylene glycol) Molecular Architecture on Particle Assembly and Ex Vivo Particle–Immune Cell Interactions in Human Blood	AI2TOX	34009935	10.1021/acsnano.1c01642
13	Interaction of gold and silver nanoparticles with human plasma: Analysis of protein corona reveals specific binding patterns	PROTCROWN	28131092	10.1016/j.colsurfb.2017.01.037
14	Protein corona fingerprinting predicts the cellular interaction of gold and silver nanoparticles	PROTCROWN	24517450	10.1021/nn406018q
15	Secreted biomolecules alter the biological identity and cellular interactions of nanoparticles	PROTCROWN	24797313	10.1021/nn4061012
16	Surface chemistry of gold nanoparticles determines the biocorona composition impacting cellular uptake, toxicity and gene expression profiles in human endothelial cells	PROTCROWN	28420299	10.1080/17435390.2017.1314036
17	In Vivo Formation of Protein Corona on Gold Nanoparticles: The Effect of Size and Shape	PROTCROWN	29292433	10.1039/C7NR08322J
18	The viral protein corona directs viral pathogenesis and amyloid aggregation	PROTCROWN	31133680	10.1038/s41467-019-10192-2
19	Identifying cell receptors for the nanoparticle protein corona using genome screens	PROTCROWN	35953550	10.1038/s41589-022-01093-5
20	Dynamic intracellular exchange of nanomaterials' protein corona perturbs proteostasis and remodels cell metabolism	PROTCROWN	35653569	10.1073/pnas.2200363119
21	An Evaluation of Blood Compatibility of Silver Nanoparticles	PROTCROWN	27145858	10.1038/srep25518
22	Protein adsorption is required for stealth effect of poly(ethylene glycol)- and poly(phosphoester)-coated nanocarriers	PROTCROWN	26878141	10.1038/nnano.2015.330
23	Characterization of the bionano interface and mapping extrinsic interactions of the corona of nanomaterials	PROTCROWN	26324751	10.1039/C5NR01970B
24	Protein source and choice of anticoagulant decisively affect nanoparticle protein corona and cellular uptake	PROTCROWN	26804616	10.1039/C5NR08196C
25	Rapid formation of plasma protein corona critically affects nanoparticle pathophysiology	PROTCROWN	24056901	10.1038/nnano.2013.181
26	Visualization of the protein corona: Towards a biomolecular understanding of nanoparticle-cell-interactions	PROTCROWN	28632260	10.1039/C7NR02977B
27	Nanoparticle Biomolecular Corona-Based Enrichment of Plasma Glycoproteins for N-Glycan Profiling and Application in Biomarker Discovery	PROTCROWN	35341249	10.1021/acsnano.1c09564
28	The timeline of corona formation around silica nanocarriers highlights the role of the protein interactome	PROTCROWN	27858044	10.1039/C6NR04765C
29	The species origin of the serum in the culture medium influences the in vitro toxicity of silica nanoparticles to HepG2 cells	PROTCROWN	28796831	10.1371/journal.pone.0182906
30	Stealth Effect of Biomolecular Corona on Nanoparticle Uptake by Immune Cells	PROTCROWN	26378619	10.1021/acs.langmuir.5b02158
31	Protein Adsorption from Biofluids on Silica Nanoparticles: Corona Analysis as a Function of Particle Diameter and Porosity	PROTCROWN	26371804	10.1021/acsami.5b07631
32	Physical-Chemical Aspects of Protein Corona: Relevance to In Vitro and In Vivo Biological Impacts of Nanoparticles	PROTCROWN	21288025	10.1021/ja107583h
33	Nanoparticle size is a critical physicochemical determinant of the human blood plasma corona: A comprehensive quantitative proteomic analysis	PROTCROWN	21866933	10.1021/nn201950e
34	Effects of the presence or absence of a protein corona on silica nanoparticle uptake and impact on cells	PROTCROWN	22721453	10.1021/nn300223w
35	Zeolite Nanoparticles for Selective Sorption of Plasma Proteins	PROTCROWN	26616161	10.1038/srep17259
36	Differential analysis of "protein corona" profile adsorbed onto different nonviral gene delivery systems	PROTCROWN	21867671	10.1016/j.ab.2011.08.003
37	Do plasma proteins distinguish between liposomes of varying charge density?	PROTCROWN	22266103	10.1016/j.jprot.2012.01.003
38	In Vivo Biomolecule Corona around Blood-Circulating, Clinically Used and Antibody-Targeted Lipid Bilayer Nanoscale Vesicles	PROTCROWN	26135229	10.1021/acsnano.5b03300
39	In vivo protein corona patterns of lipid nanoparticles	PROTCROWN	—	10.1039/C6RA25493D
40	Influence of dynamic flow environment on nanoparticle-protein corona: From protein patterns to uptake in cancer cells	PROTCROWN	28273493	10.1016/j.colsurfb.2017.02.037
41	Lipid composition: a "key factor" for the rational manipulation of the liposome-protein corona by liposome design	PROTCROWN	—	10.1039/C4RA13335H
42	The liposome-protein corona in mice and humans and its implications for in vivo delivery	PROTCROWN	32261967	10.1039/C4TB01316F
43	Nanoparticles-cell association predicted by protein corona fingerprints	PROTCROWN	27279572	10.1039/C6NR03898K
44	A proteomics-based methodology to investigate the protein corona effect for targeted drug delivery	PROTCROWN	25132011	10.1039/C4MB00292J
45	Selective targeting capability acquired with a protein corona adsorbed on the surface of 1,2-dioleoyl-3-trimethylammonium propane/DNA nanoparticles	PROTCROWN	24245615	10.1021/am404171h
46	Surface chemistry and serum type both determine the nanoparticle-protein corona	PROTCROWN	25731725	10.1016/j.jprot.2015.02.009
47	Surface coatings shape the protein corona of SPIONs with relevance to their application in vivo	PROTCROWN	23002920	10.1021/la302446h
48	Significance of surface charge and shell material of superparamagnetic iron oxide nanoparticle (SPION)-based core/shell nanoparticles on the composition of the protein corona	PROTCROWN	26218117	10.1039/C4BM00264D
49	Proteomics analysis reveals distinct corona composition on magnetic nanoparticles with different surface coatings: Implications for interactions with primary human macrophages	PROTCROWN	26444829	10.1371/journal.pone.0129008
50	The on-bead digestion of protein corona on nanoparticles by trypsin immobilized on the magnetic nanoparticle	PROTCROWN	24572545	10.1016/j.chroma.2014.01.077
51	Nanoparticle size matters in the formation of plasma protein coronas on Fe_3O_4 nanoparticles	PROTCROWN	24974013	10.1016/j.colsurfb.2014.06.016
52	Magnetic nanoparticles to recover cellular organelles and study the time-resolved nanoparticle-cell interactome throughout uptake	PROTCROWN	24737750	10.1002/smll.201303841
53	Variations in biocorona formation related to defects in the structure of single-walled carbon nanotubes and the hyperlipidemic disease state	PROTCROWN	28814800	10.1038/s41598-017-08896-w
54	Biophysics and protein corona analysis of Janus cyclodextrin-DNA nanocomplexes: Efficient cellular transfection on cancer cells	PROTCROWN	28315770	10.1016/j.bbagen.2017.03.010
55	Protein Nanoparticle Charge and Hydrophobicity Govern Protein Corona and Macrophage Uptake	PROTCROWN	33054178	10.1021/acsami.0c12341
56	Mechanistic understanding of in vivo protein corona formation on polymeric nanoparticles and impact on pharmacokinetics	PROTCROWN	28974673	10.1038/s41467-017-00600-w

Table S2. List of the 44 curated HBPs.

Gene name	UniProt ID	Protein name	Heparin-binding evidence	PMID	DOI
APOE	P02649	Apolipoprotein E	GO:0008201; IntAct portal: EBI-28951763	3947350; 26970331	10.1016/s0006-291x(86)80489-2; 10.1002/elps.201600043
VTN	P04004	Vitronectin	GO:0008201; IntAct portal: EBI-28951763	12071840; 26970331	10.1042/bj20011297; 10.1002/elps.201600043
F2	P00734	Prothrombin	GO:0008201; IntAct portal: EBI-28951763	675585; 26970331	10.1016/0049-3848(78)90269-4; 10.1002/elps.201600043
APOH	P02749	Beta-2-glycoprotein 1	GO:0008201; IntAct portal: EBI-28951763	11711550; 26970331	10.1074/jbc.m110176200; 10.1002/elps.201600043
HRG	P04196	Histidine-rich glycoprotein	GO:0008201; IntAct portal: EBI-28951763	16436387; 26970331	10.1074/jbc.m508483200; 10.1002/elps.201600043
KNG1	P01042	Kininogen-1	GO:0008201; IntAct portal: EBI-28951763	12911595; 26970331	10.1046/j.1538-7836.2003.00291.x; 10.1002/elps.201600043
CFH	P08603	Complement factor H	GO:0008201; IntAct portal: EBI-25436079	1832158; 17339482	10.1016/s0021-9258(18)55379-5; 10.4049/jimmunol.178.6.3831
SERPINC1	P01008	Antithrombin-III	GO:0008201; IntAct portal: EBI-28951763	6693405; 26970331	10.1016/s0021-9258(17)43548-4; 10.1002/elps.201600043
GSN	P06396	Gelsolin	Manual curation	16475811; 21348501	10.1021/bi0519295; 10.1021/bi101905n
CLU	P10909	Clusterin	IntAct portal: EBI-28951763; Manual curation	9537999; 26970331	10.1021/bi972367v; 10.1002/elps.201600043
APOB	P04114	Apolipoprotein B-100	GO:0008201; IntAct portal: EBI-28951763	3301850; 26970331	10.1016/s0021-9258(18)60931-7; 10.1002/elps.201600043
C4BPA	P04003	C4b-binding protein alpha chain	IntAct portal: EBI-28951763	2136880; 26970331	10.4049/jimmunol.144.1.204; 10.1002/elps.201600043
F11	P03951	Coagulation factor XI	GO:0008201; IntAct portal: EBI-28951763	11412111; 26970331	10.1021/bi0027433; 10.1002/elps.201600043
CLEC3B	P05452	Tetranectin	GO:0008201; IntAct portal: EBI-28951763	10727405; 26970331	10.1042/bj3470083; 10.1002/elps.201600043
FN1	P02751	Fibronectin	GO:0008201; IntAct portal: EBI-28951763	10075919; 26970331	10.1093/emboj/18.6.1468; 10.1002/elps.201600043
SERPINA5	P05154	Plasma serine protease inhibitor	GO:0008201; IntAct portal: EBI-28951763	18974053; 26970331	10.1074/jbc.m805974200; 10.1002/elps.201600043
C1QC	P02747	Complement C1q subcomponent subunit C	Manual curation	20548024	10.4049/jimmunol.1000184
C1QB	P02746	Complement C1q subcomponent subunit B	IntAct portal: EBI-28951763; Manual curation	20548024; 26970331	10.4049/jimmunol.1000184; 10.1002/elps.201600043
F10	P00742	Coagulation factor X	Manual curation	10652320	10.1074/jbc.275.5.3320
C4B	P0C0L5	Complement C4-B	IntAct portal: EBI-28951763; Manual curation	16125850; 26970331	10.1016/j.bbagen.2005.08.003; 10.1002/elps.201600043
SAA1	P0DJI8	Serum amyloid A-1 protein	GO:0008201	10066777	10.1074/jbc.274.11.7172
SERPING1	P05155	Plasma protease C1 inhibitor	IntAct portal: EBI-28951763	17488724; 26970331	10.1074/jbc.m700841200; 10.1002/elps.201600043
PF4	P02776	Platelet factor 4	GO:0008201; IntAct portal: EBI-28951763	9531587; 26970331	10.1182/blood.v91.8.2772.2772_2772_2780; 10.1002/elps.201600043
C1QA	P02745	Complement C1q subcomponent subunit A	Manual curation	20548024	10.4049/jimmunol.1000184
COMP	P49747	Cartilage oligomeric matrix protein	GO:0008201	17588949	10.1074/jbc.m611390200
SERPIND1	P05546	Heparin cofactor 2	GO:0008201	6895893	10.1016/s0021-9258(18)34900-7
F9	P00740	Coagulation factor IX	Manual curation	12397068	10.1074/jbc.m208485200
PF4V1	P10720	Platelet factor 4 variant	GO:0008201	23536183	10.1074/jbc.m113.455329
PPBP	P02775	Platelet basic protein	Manual curation	28245630	10.3390/ijms18030508
C4A	P0C0L4	Complement C4-A	Manual curation	16125850	10.1016/j.bbagen.2005.08.003
S100A9	P06702	Protein S100-A9	IntAct portal: EBI-28951763; Manual curation	11723110; 26970331	10.1074/jbc.m102950200; 10.1002/elps.201600043
CFB	P00751	Complement factor B	Manual curation	16125850	10.1016/j.bbagen.2005.08.003
LBP	P18428	Lipopolysaccharide-binding protein	Manual curation	15699163	10.4049/jimmunol.174.4.2280
C8A	P07357	Complement component C8 alpha chain	Manual curation	16125850	10.1016/j.bbagen.2005.08.003
C4BPB	P20851	C4b-binding protein beta chain	Manual curation	2136880	10.4049/jimmunol.144.1.204
C9	P02748	Complement component C9	Manual curation	16125850	10.1016/j.bbagen.2005.08.003
C8G	P07360	Complement component C8 gamma chain	Manual curation	16125850	10.1016/j.bbagen.2005.08.003
HSPA8	P11142	Heat shock cognate 71 kDa protein	IntAct portal: EBI-28964410; Manual curation	7548155; 26970331	10.1016/0167-4838(95)00113-9; 10.1002/elps.201600043
LTF	P02788	Lactotransferrin	GO:0008201; IntAct portal: EBI-28951763	8089135; 26970331	10.1016/s0021-9258(17)31566-1; 10.1002/elps.201600043
C8B	P07358	Complement component C8 beta chain	Manual curation	16125850	10.1016/j.bbagen.2005.08.003
CFD	P00746	Complement factor D	IntAct portal: EBI-28951763; Manual curation	8487783; 26970331	10.1016/0161-5890(93)90079-q; 10.1002/elps.201600043
C6	P13671	Complement component C6	Manual curation	16125850	10.1016/j.bbagen.2005.08.003
ITGB3	P05106	Integrin beta-3	Manual curation	16982628	10.1074/jbc.m605291200
APOA5	Q6Q788	Apolipoprotein A-V	GO:0008201	17326667	10.1021/bi7000533

References

[1] Canchola, A.; Li, K.; Chen, K.; Borboa-Pimentel, A.; Chou, C.; Dela Rama, R.; Chen, C.-Y.; Chen, X.; Strobel, M.; Riviere, J. E.; et al. Meta-analysis and machine learning prediction of protein corona composition across nanoparticle systems in biological media. *ACS Nano* 2025, 19 (43), 37633-37650.

[2] Su, Y.; Fu, X.; Wei, L.; Zhang, Z.; Zou, Q.; Cui, F. PROTCROWN: A Manually Curated Resource of Protein Corona Data for Unlocking the Potential of Protein–Nanoparticle Interactions. *Nano Lett.* 2025, 25 (5), 1739-1744.

[3] Ashburner, M.; et al. Gene ontology: tool for the unification of biology. *Nat Genet.* 2000 25 (1), 25-29.

[4] The Gene Ontology Consortium. The Gene Ontology knowledgebase in 2026. *Nucleic Acids Res.* 2025, D1779-D1792.

[5] Orchard, S.; et al. The MIntAct project—IntAct as a common curation platform for 11 molecular interaction databases. *Nucleic Acids Res.* 2014, D358-D363.
